# Data Extraction of Circular-Shaped and Grid-like Chart Images

**DOI:** 10.3390/jimaging8050136

**Published:** 2022-05-12

**Authors:** Filip Bajić, Josip Job

**Affiliations:** 1 University Computing Centre, University of Zagreb, 10000 Zagreb, Croatia; 2 Faculty of Electrical Engineering, Computer Science and Information Technology Osijek, 31000 Osijek, Croatia; josip.job@ferit.hr

**Keywords:** chart data extraction, chart image processing, data visualization, image processing and computer vision

## Abstract

Chart data extraction is a crucial research field in recovering information from chart images. With the recent rise in image processing and computer vision algorithms, researchers presented various approaches to tackle this problem. Nevertheless, most of them use different datasets, often not publicly available to the research community. Therefore, the main focus of this research was to create a chart data extraction algorithm for circular-shaped and grid-like chart types, which will accelerate research in this field and allow uniform result comparison. A large-scale dataset is provided containing 120,000 chart images organized into 20 categories, with corresponding ground truth for each image. Through the undertaken extensive research and to the best of our knowledge, no other author reports the chart data extraction of the sunburst diagrams, heatmaps, and waffle charts. In this research, a new, fully automatic low-level algorithm is also presented that uses a raster image as input and generates an object-oriented structure of the chart of that image. The main novelty of the proposed approach is in chart processing on binary images instead of commonly used pixel counting techniques. The experiments were performed with a synthetic dataset and with real-world chart images. The obtained results demonstrate two things: First, a low-level bottom-up approach can be shared among different chart types. Second, the proposed algorithm achieves superior results on a synthetic dataset. The achieved average data extraction accuracy on the synthetic dataset can be considered state-of-the-art within multiple error rate groups.

## 1. Introduction

In the world of advanced Internet technologies, data and information have a significant role. The data accumulate exponentially, and it is difficult to distinguish and notice between important information and irrelevant information. When it comes to data that are represented by numbers, which can be piled up and unstructured or organized in tables, the critical information is not easily readable. It often requires mental effort and previous knowledge in the given field. Chart images are used to make data more readable by the individual, easier to understand, and easier for information transfer.

Data visualization (chart images) are graphs or diagrams used to present the tabular data’s quantitative information. These images have been used since the 18th century [1] in different science fields, including mathematics, statistics, and analytics. Today, chart images prevail in scientific papers, financial reports, textbooks, webpages, and news articles [2]. Well-designed chart images are time-consuming to produce and often require additional effort from the author, and even then, these data representations are not easily accessible by everyone. The problem affects people as well as machines. For example, people with impaired vision and all blind individuals can not access the “locked” information inside chart images. These people often rely on assistive technology (braille display, screen readers, and speech converters), which can only read the information that is provided by the author in the surrounding text [3,4]. Manual reading of chart images is often inaccurate and can not be used for scientific purposes. The underlying data can also be necessary to all individuals for different general purposes, including verification or comparison of the achieved results.

On the other hand, while most people can read and decode chart images, machines can not easily approach them. For example, the results of the Internet search engines can not include information from chart images. The programs for comparing, re-using, or transmuting chart images are nonexistent or hardly available. In order to solve the challenges mentioned above, many authors contributed to the field of reverse-engineering chart images.

When reverse-engineering of chart images are discussed, image classification image processing should be noted. Image classification is a well-studied process in machine learning which refers to categorizing and labeling images according to the visual content. Over the years, authors used different key approaches to achieve the highest average classification accuracy. These approaches can be grouped into four categories: custom algorithm [5,6,7,8], model-based approach [9,10,11], Support Vector Machines [2,12,13], and neural networks [14,15,16,17].

After classifying an image, the next step is to decompose that image into graphical and textual data. Text localization, text processing, and text recognition are well-known challenges addressed using Optical Character Recognition (OCR) systems. This research uses a publicly available out-of-the-box OCR system whose output result quality is not this research topic.

### Research Contributions

In this work, we presented a new, fully automatic low-level algorithm that uses a raster image as input and generates an object-oriented structure of the chart of that image. The main novelty of the proposed approach is in chart processing on binary images instead of commonly used pixel counting techniques. Our primary contributions can be summarized as follows:

- A novel algorithm for processing chart images that is based on processing binary images. The proposed novel algorithm follows a bottom-up approach and uses pixel-level, and many low-level operations and procedures on pie charts, donut charts, sunburst diagrams, heatmaps, and waffle charts. The sunburst diagrams, heatmaps, and waffle charts, to our knowledge, have never been processed before, and we are the first to present state-of-the-art results;
- A large-scale dataset containing 120,000 chart images is organized into 20 categories. Each image in the dataset is labeled and joined with additional documentation that includes the ground truth. This dataset is publicly available at figshare: https://doi.org/10.6084/m9.figshare.19524844.v1 (accessed on 13 April 2022);
- An extensive experimental analysis of the proposed algorithm on the created dataset and commonly used International Conference on Document Analysis and Recognition (ICDAR) dataset to provide a valid comparison.
- The proposed image classification system is related to our previous research, where Siamese Convolutional Neural Network achieved state-of-the-art results, 100% average classification accuracy, and an F-1 score over seven data visualization types.

The remainder of the paper is organized in the following manner. Section 2 presents the current research in chart data extraction. Section 3 provides information about the proposed algorithm and a created dataset. In Section 4, an evaluation of the proposed algorithm is conducted on synthetic chart images and real-world chart images. The discussion of our findings and open challenges are presented in Section 5. Finally, Section 6 gives the final remarks on this experiment and provides plans for future research.

## 2. Related Work

The two primary goals of authors who deal with data extraction of chart images are to obtain the original data table from which the visualization has been created or obtain the used visual styles (color map, fonts, dimensions, and position of elements). Through the years in chart data extraction, authors used different data recovery approaches that can be grouped into two categories: the automatic and the interactive. In the automatic approach, the user inputs a chart image into the system, and the system decodes the image and returns an object. This type is in the majority and requires no additional interaction or correction of the user. On the other hand, in the interactive approach, it is expected for the user to click or select the area of interest from which the data should be exported. The required selections are often starting and ending points or a group of points. These systems are often more complex than the automatic ones and are only researched in ChartSense [18] and by Yang et al. [19]. While interactive systems can achieve competitive results, they are prone to error due to required human interaction. Another drawback is the required time for processing large-scale datasets.

Compared to chart type classification, chart data extraction supports considerably fewer chart types. The most processed chart types are bar, line, and pie chart. Scatter plot is researched in [20,21], and area and radar chart in ChartSense [18]. The developed algorithms often rely on low-level knowledge, and the diversity of chart types creates further challenges. The algorithms expect to find chart objects in predefined places and use the combination of pixel distance and scaling values. The axes (the finite number of black pixels in a row) are obligatory for bar and line charts. On one side of the axes are labels that OCR detects, and on the other side are chart objects detected by pixel color change. Since pie charts do not contain axes, different methods are used. With the usage of Random sample consensus (RANSAC) regression, a color change between adjacent pixels can be detected, which translates to a pie slice, ReVision [22], ChartSense [18], and Chart-Text [23]. Another method for detecting pie slices is counting the number of colored pixels between two boundaries [14,24].

Deep learning models can also detect the objects in chart images. Liu et al. use the Recurrent Neural Network (RNN) for detecting angles in pie charts. The developed neural network achieves bar data extraction of 79.40% and pie data extraction of 88.00% [25]. Choi et al. in Visualizing for the Non-Visual use textual and graphical information for data extraction [14]. Three different algorithms are created for three chart types: bar, line, and pie. The objects are detected using different object detection models (Yolo2, Faster R-CNN, SSD), and bounding boxes are drawn. By using different image processing techniques inside the bounding boxes, pixel values are obtained. Combining neural networks and low-level operations results in data extraction accuracy for bar charts 89–99%, line charts 72–88%, and pie charts 86–92% (the results depend on the used dataset). The state-of-the-art pie chart data extraction results are found in [24]. Here, the authors developed a novel image processing-based algorithm that works with 2D and 3D pie charts. The salt-and-pepper noise is removed from the input image, the image is converted to a grayscale image, and after the gradient analysis, the image is binarized. This algorithm is also considered low-level as it counts the number of pixels in the area of interest. The reported accuracy for 2D pie charts is 99%, while for 3D pie charts, 97%. A comparison with other state-of-the-art papers can not be made, as this is the only algorithm that works with 2D and 3D pie charts. A summary of the presented related work is summarized in Table 1. As seen in Table 1, not all authors report comparable data extraction results or dataset size. The last column, “Dataset,” is a sum of all images used for chart data extraction. From the evaluation point reported in the papers, it is hard to notice what actual number of images were used for chart data extraction, as the dataset often consists of images from different sources.

## 3. Methodology

In this section, details about existing datasets of chart images are provided. The availability and accessibility of these datasets are discussed. The details about our dataset are given. At the end of the section, the core of the proposed algorithm is presented, and the difference between circular-shaped chart images and grid-like chart images pre-processing is explained.

### 3.1. The Dataset

Chart data extraction requires chart datasets for training and testing. Creating labeled chart datasets is often a complex and time-consuming process. The majority of the papers in the provided Table 1 use small, often private, datasets. Some of the presented papers created a dataset and made it publicly available. Due to the time between the release of a paper containing a dataset and the time of writing this article, most of the datasets are no longer available. Some of the datasets are inaccessible due to restricted access, and some datasets are no longer complete. Each of these datasets has a limited number of chart types and images, which is often inadequate for chart image processing tasks. The detailed ground truth consisting of all parameters used in chart image creation is also unavailable. In order to solve the limitations mentioned above of existing datasets, we publicly provide our dataset. The comparison of related datasets is presented in Table 2. The provided datasets in Table 2 are not only from papers dealing with chart data extraction but also from research dealing with chart image classification and chart text processing.

To our knowledge, the datasets from ICDAR [35] and International Conference on Pattern Recognition (ICPR) [36] are fully available and accessible. These datasets are used in Competition on Harvesting Raw Tables (CHART). Their sub-tasks are chart type classification, text detection and recognition, text role classification, axis analysis, legend analysis, and chart data extraction. The images are not labeled nor organized by chart type, but ground truth and annotation are provided in a separate file. This file is not easily accessible and requires further processing to acquire a human-readable object-oriented format. Because of the diversity of the chart images, only pie and donut charts from these datasets are used for comparison purposes. The heatmaps can not be compared, as our algorithm requires a heatmap that consists of rows and columns. There are no available datasets for comparison for the sunburst diagrams and waffle charts.

Encouraged by the challenges mentioned above, we developed a dataset containing chart images that are fully organized and labeled. Each image in the dataset is also supported by a text document with an object-oriented structure containing the ground truth and metadata. The images are in “.png” format, and the resolution is 1366 × 768 pixels. The dataset is entirely made with Plotly [37], and the total size is 5 GB. All images are organized into two categories. The first category includes training and testing data. The testing data category does not contain any images from training data. Each category can be further split into simple and complex images for each chart type. The dataset split containing simple images, e.g., pie chart, contains only pie charts whose number of pie slices is less or equal to three. These images also always contain a title and a legend. The complex split contains pie charts with up to 15 pie slices where title and legend are not obligatory. In other words, the simple split contains everything required for a human to understand and read the chart image, while a complex split often omits some elements and has a more significant number of data points. The numerical values, colors, and texts are randomly generated for all chart types. In Table 3. the details of our dataset are provided.

### 3.2. The Algorithm

**Figure 1:**
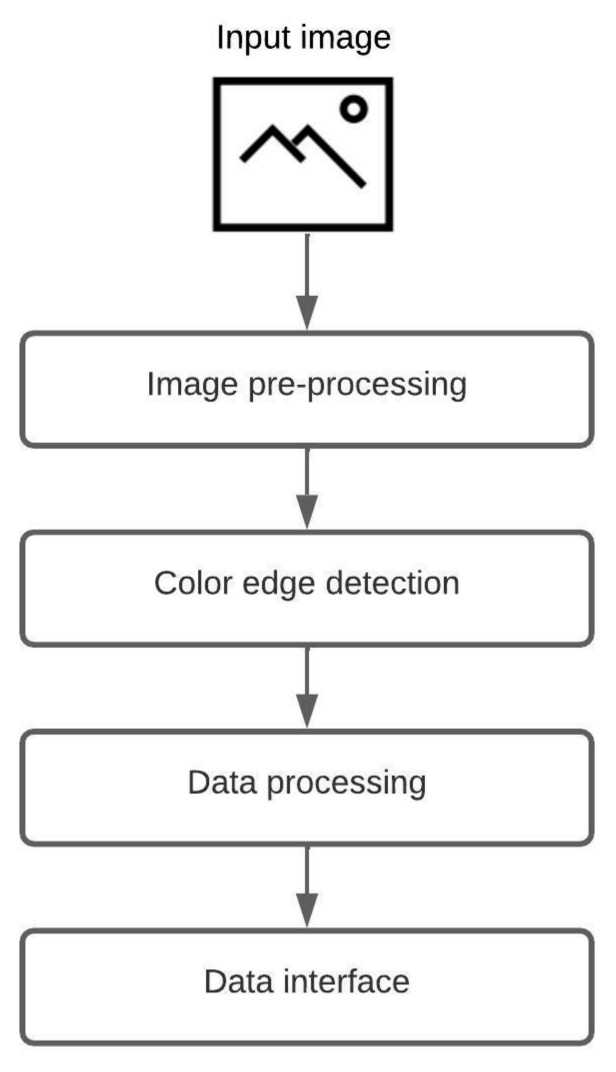
The core process of the proposed algorithm.

A fully automatic low-level algorithm that accepts a raster image as input and generates an object-oriented structure of the chart in the image is proposed. The system supports two groups of chart images, where one group consists of circular-shaped charts (pie chart, donut chart, and sunburst diagram), and the other group includes grid-like charts (heatmap and waffle chart). Since chart images can have a diversity of applied styles, a few restrictions are made. First, the input image should not contain a 3D version of a chart nor any 3D effects (e.g., shadows, highlights). Second, the input image can not include multiple charts. Third, all chart components should have a distinct color. An illustration of the proposed algorithm is shown in Figure 1. The algorithm consists of four steps: image pre-processing, color edge detection, data processing, and data interface. The majority of the proposed process is the same for all five chart types, including pie charts, donut charts, sunburst diagrams, heatmaps, and waffle charts, and the only difference is in the third step, data processing.

The first step of the displayed algorithm is image pre-processing. Image pre-processing is the most crucial step that deals with image preparation and manipulation. The main task of this step is to prepare the input image for future analysis and chart data extraction. Three filters are applied over the input image. The first filter is used for image color space normalization (blue-green-red color space). The second filter reduces possible noise in the image, which can be salt-and-pepper or amplifier noise (median blur and Gaussian blur). The third filter makes the transition between adherent regions in the image more obvious (sharpen kernel). The pixel brightness increases, and the edges between the distinct colors are emphasized. This filtered image is supplied to Keras OCR v0.8.9, where text elements are detected. After the detection, the two images are created, one with textual elements and the other with graphical elements. The graphical image is then converted to a grayscale image to reduce details on the image.

The second step, color edge detection, uses the image with graphical elements from the first step. The proposed algorithm scans image for unique colors. For each detected unique color, a mask is created. The mask activates (logical 1) all pixels matching the unique color and deactivates (logical 0) all other pixels. In the end, all created masks are joined, and the narrow border of one pixel is present, indicating a border between adherent regions.

In the third step, data processing, all calculations are carried out. Each chart type can have numerous variations in applied styles and shapes. The color scheme and aspect ratio of a chart vary across all images. Due to the difference in style and design, two different data processing algorithms are required, one for circular-shaped chart images and the other for grid-like chart images. More information about data processing is provided in the following sections.

The acquired information is labeled, grouped, and organized in the last step, the data interface. The system exports an object-oriented structure that other applications of interest can consume. An example of a data interface for the sunburst diagram and heatmap is provided in the following sections.

#### 3.2.1. Pie, Donut, and Sunburst

**Figure 2:**
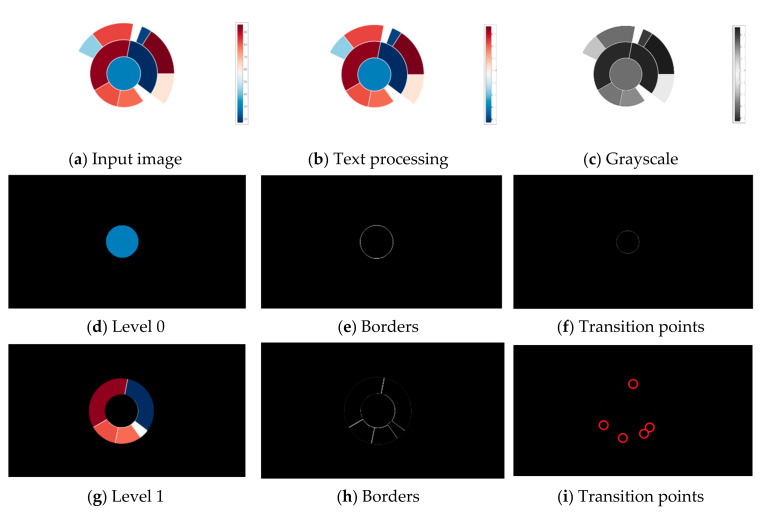

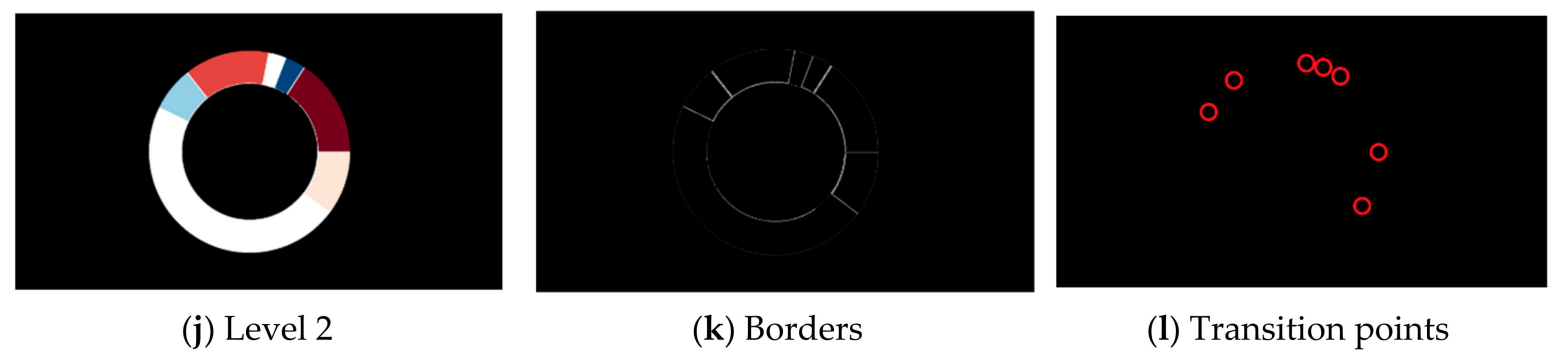
Processing of sunburst diagram. From input image (**a**), the text is removed (**b**), and the image is converted to grayscale (**c**). In this example, the input image is divided into three levels; the most inner circle is “Level 0” (**d**), the middle circle is “Level 1” (**g**), and the most outer circle is “Level 2” (**j**). Each level (or circle) contains unique colors whose borders are shown in (**e**,*h*,**k**). With borders detected, a transition points can be obtained. The transition points are exact coordinates where the pixel changes color value (**f**,**i**,**l**).

Image pre-processing is performed to extract data from circular-shaped chart types (pie charts, donut charts, or sunburst diagrams). Input raster image Figure 2a is filtered, and all text is removed, Figure 2b. The image containing only graphical elements is converted to grayscale, Figure 2c. Over the grayscale image, a Hough Transform for circle detection is used. Hough Transform extracts features from an image and then, using a voting procedure, determines the shape of the objects present in an image. The Hough Transform is performed until no new circles are detected. With the number of detected circles, a chart type can be determined. If only one circle is detected, the input image contains a pie chart. A donut chart is detected if two circles are detected where the one closest to the center does not contain any color. In case of detecting two or more circles, the sunburst diagram is presented.

In order to demonstrate a proposed algorithm, a sunburst diagram from the complex dataset is used. For each circle in the diagram, a mask is created. The mask deactivates all pixels not related to a selected circle (Figure 2d,g,j). By creating a mask for each unique color, a border between adherent regions (slices) is detected, Figure 2e,h,k. The detection of transition points is carried out in the last step. The transition points are exact coordinates where the pixel changes color value. An additional circle is drawn inside the created mask to obtain these coordinates. The location where this circle crosses a border is considered a transition point. The transition point is a size of one pixel, and a bright red circle is drawn around it to make it more noticeable (Figure 2f,i,l).

**Figure 3:**
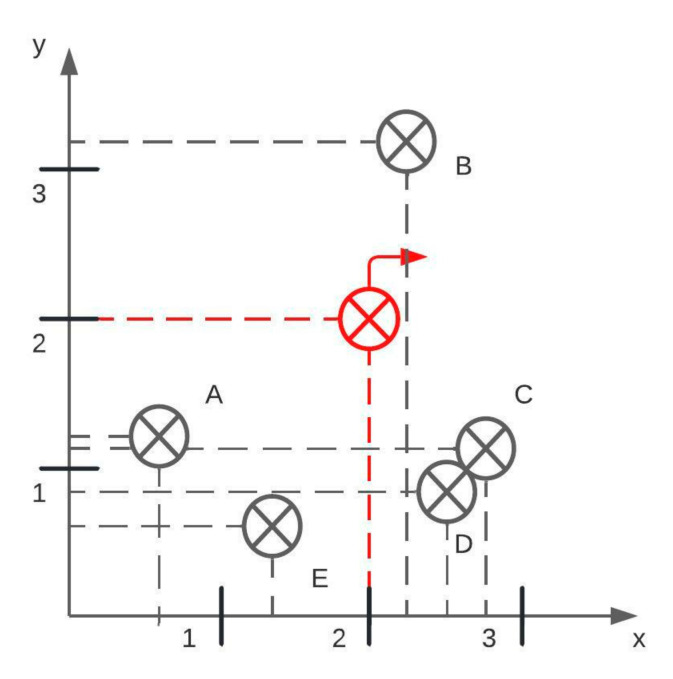
An example of reading transition points in a clockwise direction. The red transition point is the center, and the correct output is [A, B, C, D, E]. These transition points are from Figure 2i.

When obtaining an (x, y) pixel value from transition points, its placement in the image should be preserved. If the upper left corner in the raster image is considered (0, 0), the transition points are obtained by scanning the image from left to right and top to bottom. The result is an array with transition points incorrectly placed. Consider a Cartesian coordinate system with points placed as in Figure 2i and Figure 3. When scanning an image for points, the result is: [B, A, C, D, E]. According to the array, point A is surrounded by neighbor points B and C, which is incorrect as the actual neighbor points are B and E. In order to preserve a natural placement of points, a center point is defined, and all other points are sorted clockwise direction. The center point in Figure 3 is colored in red, and in the chart image, it is the center of the circle detected with the Hough Transform. With obtained points of interest, a slope *m* is calculated using formula (1):

(1) $$m=\frac{y_{2}-y_{1}}{x_{2}-x_{1}}$$

where (*x*_1_, *y*_1_) are center point coordinates, and (*x*_2_, *y*_2_) are coordinates of a transition point. In order to calculate an angle between two points, formula (2) is used:

(2) $$\theta =arctan\;\left(m\right)\;$$

where θ is the angle. The last needed parameter for calculating the area of a sector is distance *d*. The distance formula between two points is (3):

(3) $$d=\sqrt{[{\left(x_{2}-x_{1}\right)}^{2}+[{\left(y_{2}-y_{1}\right)}^{2}]}$$

With all parameters calculated, an area of sector *P* is (4):

(4) $$P=\frac{\theta }{360}\times \pi \times d^{2}$$

**Figure 4:**
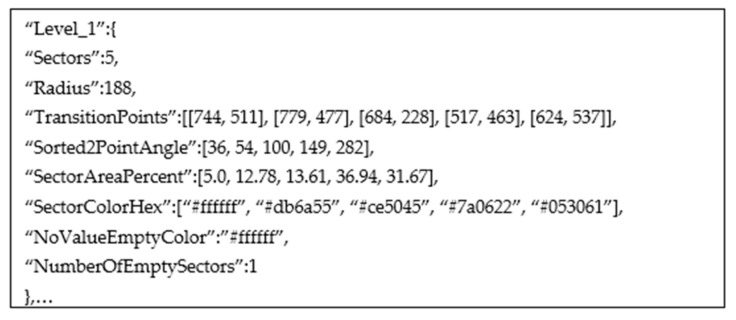
An example of data interface for Figure 2g.

In the case of sunburst diagrams, all calculated angles are laid out on the circle to decode the relation between a parent and a child slice. All angles included within two angles are considered child angles which translate to child slices. To better explain the relations, consider Figure 2d,g. Figure 2d does not include any transition point which indicates a full circle or 360° angle. Figure 2g has five angles, whose values are shown in Figure 4. Values of the outer circle are compared to the values of the inner circle. Since the inner circle is a full circle, and all outer angles are less than 360°, all pie slices belong to the inner circle, or in other words, all outer pie slices are child slices of the inner parent circle.

**Figure 5:**
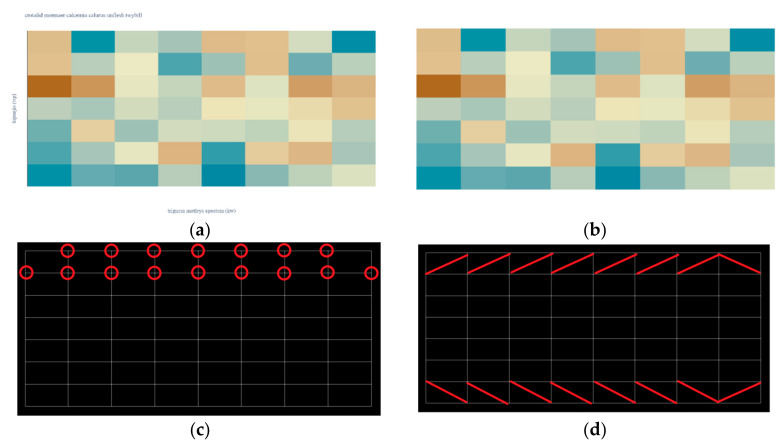
Processing of heatmap. (**a**) Input image. (**b**) Text processing. (**c**) Transition points. (**d**) Color detection.

An example of a data interface for Level 1 of Figure 2g is shown in Figure 5. The “NoValueEmptyColor” attribute is determined by comparing the background color of the chart image with the detected color of a slice. If these two colors match, the slice is empty, or in other words, the slice does not contain important information.

#### 3.2.2. Heatmap and Waffle

The heatmaps and waffle charts are almost the same from the visual style perspective. The significant difference is between two adherent regions in the border (or space). In both chart types, the regions are described by distinct colors and a rectangular shape.

Since heatmaps are often more complex than waffle charts, a heatmap from a complex dataset is used to demonstrate a proposed algorithm. The first part of the algorithm is always the same. The input raster image Figure 5a is filtered, and all text is removed, Figure 5b. The mask is created for each unique color leaving a one-pixel border between adherent regions (rectangles), Figure 5c. Since these borders are part of a grid system, the color transition points can easily be determined. In Figure 5c, a bright red circle is drawn around the first row’s transition points.

**Figure 6:**
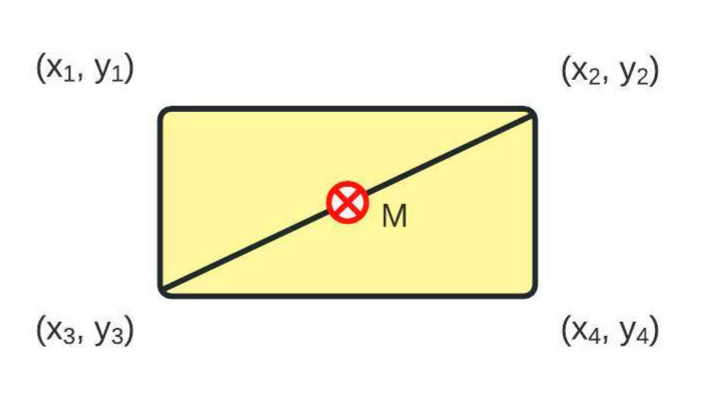
The calculation of midpoint *M.* The midpoint is used in Figure 5d for color detection.

Compared with the algorithm for a pie chart, donut chart, and sunburst diagram, the reading of the coordinate values of points is always in the correct order (left to right, then top to bottom). In order to acquire a color code of the area of interest, a simple midpoint calculation is performed following the scheme from Figure 6. The midpoint *M* is calculated using (5):

(5) $$M=\left(\frac{x_{3}+x_{2}}{2},\;\frac{y_{3}+y_{2}}{2}\right)$$

where (*x*_1_, *y*_1,_ *x*_2_, *y*_2,_ *x*_3_, *y*_3,_ *x*_4_, *y*_4_) are coordinate points that describe a rectangular shape. The midpoint color is equal to the color of the area of interest. In Figure 5d, the points used for calculating the midpoint are connected with a bright red line. The last area of interest in a row and the last row of the heatmap are always inverted since the corners of the heatmap do not include a single transition point.

**Figure 7:**
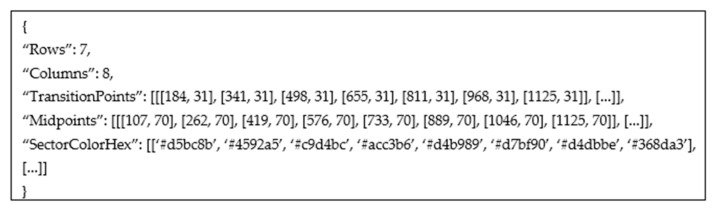
An example of data interface for transition points in the first row of Figure 5c.

An example of a data interface for Figure 5a is shown in Figure 7. The output contains only the points for the first row. Note that the number of transition points is seven, following Figure 5c. The number of midpoints is eight, following Figure 5d and matching the detected number of columns in the heatmap.

#### 3.2.3. Legend Detection

**Figure 8:**
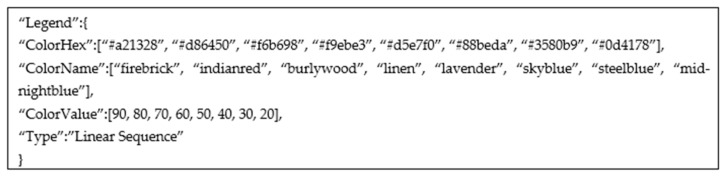
An example of data interface for legend processing of Figure 2a.

The legend detection is based on the results of the processed graphical image. The used style elements in chart data extraction belong to the chart type. The rest of the unprocessed colored elements in the image is considered to be a legend. The legend can be anywhere in the chart surrounding but can not be a part of a chart (e.g., overlapping legend and chart elements). The legend can also come in different shapes (markers or color scales) and different orientations (horizontal or vertical). The detected color elements are matched with the closest text boxes from Keras OCR. Each text box is separately processed, and string or numerical values are detected. If a string value is detected, the algorithm looks for nearby text boxes where the distance between the two is less than 10 pixels [30]. The nearby text boxes are grouped, resulting in a multiword label. In the case of numerical values, the algorithm tries to parse all obtained values and return a type of sequence: linear, quadratic, cubic, or other. The legend detection algorithm is the same for a pie chart, donut chart, sunburst diagram, heatmap, and waffle chart. In Figure 8, an example of parsed legend is shown. The parsed legend is from Figure 2a.

## 4. Chart Data Extraction Evaluation

In order to evaluate the performance of a proposed algorithm on selected chart types, a method similar to ChartSense [18] and later adopted by Choi et al. [14] and Zhou et al. [31] is used. All experiments are performed on the same computer and in the same programming environment. The experiments were performed with the proposed dataset and with the ICDAR dataset. From the ICDAR dataset, only pie charts and donut charts are used. For each chart type and from each dataset, 100 images are randomly selected. The images that do not contain a legend are automatically excluded.

The method evaluates an algorithm based on the success rate. The success rate is the proportion of correctly extracted chart images and the total number of chart images in the category. The chart image is only successfully extracted if its error rate (err) is within a set threshold. The chart data accuracy is calculated with (6):

(6) $$\frac{\left|g_{i}-p_{i}\right|}{g_{i}}\;\leq \;\varepsilon$$

where *g_i_* is the ground truth of the element, *p_i_* is the calculated value of that element, and ε is an error threshold value. The threshold controls the quality of exported chart data. A lower threshold value results in higher chart data extraction accuracy. If the chart data extraction is within the set threshold value, the image is considered correctly extracted; otherwise, the image is not correctly extracted. The average accuracy *aa* is then calculated with (7):

(7) $$aa=\frac{N_{c}}{N_{t}}\;\left[\%\right]$$

where *N_c_* is the number of correctly extracted images, and *N_t_* is the total number of images in the category (*N_t_* = 100). In other words, the threshold value < 0.05 corresponds to an error rate of 5%.

The number of images that correspond to a specific error rate group is shown in Table 4 and Table 5.

The authors from related work, ChartSense [18], Choi et al. [14], and Zhou et al. [31], mostly report chart data extraction results for ε = 0.05. Following this rule, the demonstrated method in this paper correctly extracts 99% of the information from five chart types. A direct comparison of the proposed method and results from the related work can not be made as the used methods and datasets are entirely different, and their comparison could be considered as comparing apples to oranges. If looking only at pie chart data extraction accuracy, the proposed algorithm achieves 98% for ε = 0.05, Table 6. To our knowledge, no other author reported extraction results for ε = 0.0125. From the design perspective, the pie chart, donut chart, and sunburst diagram share many similarities, and thus their extraction data accuracy is similar. While the heatmaps and waffle charts share the same grid-like design, the spacing between rows and columns of the waffle chart creates incorrect recognition of the area of interest, impacting data extraction accuracy. When comparing the complexity of the proposed algorithms, the heatmaps require less image pre-processing and thus achieve a higher data extraction accuracy. Similar results are achieved with the ICDAR dataset, as shown in Table 5. Both datasets are synthetic and share a similar design, one created with Python Plotly v5.8.0 and the other with Python Matplotlib.

Additional data collection is performed with a Google image search to test the proposed algorithm on real-world chart images. The newly created dataset consists of 20 images for each chart category. The collected images are manually filtered. When searching, e.g., heatmap, the search returns many different chart interpretations and images not related to the field of study. The selected images are only the charts with the closest design to the synthetic dataset. The selected image also includes numeric information, essential for comparison purposes. For evaluation, the same method is used as for the synthetic dataset. The findings are reported in Table 7.

When looking at the ε = 0.05, the average chart data extraction is decreased to 54%, or in other words, every other image is successfully extracted. The decreased average accuracy indicates that a difference from the design perspective exists between the synthetic dataset and real-world chart images. The decreased performance is expected as the proposed algorithm is optimized for synthetic images with minimum noise and only essential chart elements. From Table 7, it can also be noted that 38% of tested chart images were not within any threshold value. In these cases, the algorithm could not recognize the borders and transition points between the areas of interest. Sunburst diagrams (40%) and waffle charts (35%) have the lowest data extraction accuracy, but their design is also the most dependent on the author. When calculating the midpoint of the heatmaps and waffle charts, the image noise impacts the results. The pixel color values are not equal, and differences exist that the human eye cannot see. Instead of taking the value of a single pixel, an average value of the pixel neighborhood should be considered. The pixel neighborhood typically consists of 4-connected or 8-connected pixels. The connected component analysis plays an important role in processing real-world images [38,39]. The small dataset size could also impact the results, as this dataset is five times smaller than the dataset with synthetic chart images. The achieved results on real-world chart images suggest that there is room for improvement of the proposed algorithm, which we will address in future work.

## 5. Discussion

The demonstrated algorithm is proven effective, but some limitations still exist. An apparent limitation of the method is text processing. Text processing (or text removal) is a crucial part of the proposed algorithm and also often the weakest point. This is due to the use of an out-of-the-box OCR engine. Therefore, the quality of text processing is not discussed in this work. In most cases, when text is simple and limited to one word, the OCR engine detects the text with high confidence. In cases when the text element is across a darker colored background or crosses the border between two areas of interest, the part of the text is missing, or the whole text element is skipped, and manual interaction is required. The observed situation is where OCR works as intended, without any recognition limitations when evaluating the proposed algorithm. OCR engines such as Google Tesseract, Microsoft OCR, or Amazon Rekognition can be tested to improve the quality of text processing. Although these engines are trained on many images, they still may end with numerous recognition limitations on chart images. Future research could examine the training of an open-source OCR engine specifically on chart images since the text elements are of different sizes, orientations, and font styles.

When evaluating the circular-shaped chart images, the whole process depends on the Hough Transform, which requires a lot of fine-tuning. The Hough Transform’s main advantage is detecting partial circles, which are a standard part of sunburst diagrams. An apparent limitation of the Hough Transform is that it can not detect multiple circles with the same center from the initial image scan; instead, multiple image scanning is required. Another limitation involves the thickness of the borders. If the circle border is thick, the Hough Transform can detect two borders (inner and outer), impacting circle detection, and additional validations are required. Small circles and small pie slices with similar color schemes can go undetected in the grayscale image, as they might be accidentally merged with neighboring areas. In order to deal with this problem, the colored image and grayscale image should be compared for the number of circles and number of slices. Instead of using the Hough Transform, the Convolutional Neural Network (CNN) can be trained to detect circles and draw a bounding box around detected ones. The bounding box would have the same center as a circle, and the distance from the center to the border would be equal to the circle’s radius.

Another challenge is seen in the grid-like chart images. In case when two adherent areas of interest share the color code, the border between the areas and the transition points are not recognized. By conducting additional calculations, the borders can be artificially created as long as the first and last row positions are known and the dimension (height and width) of one area of interest. The CNNs can also be trained to detect colored regions, significantly reducing the number of required calculations.

The results provide an excellent fit to the synthetic dataset, but the quality can be enhanced by providing an additional dataset of real-world chart images. The borders and transition points are detected only in the subset of real-world chart images. Without these elements, further calculations can not be made; thus, data extraction can not be achieved. The proposed algorithm is flexible, and many parameters can be fine-tuned. The higher number of chart images of the same type but with different designs can be covered by the number of changeable parameters. Building a suitable dataset is required to adopt the proposed algorithm better on real-world chart images. The real-world dataset of chart images would also benefit other areas of chart exploration, such as chart classification, chart text processing, and chart description generation.

To summarize, the recommendations for future studies are:

1. Training open-source OCR engine on chart images;
2. Implementation of CNN for region detection;
3. Quantitative comparison of a proposed algorithm with the algorithm that uses CNN;
4. Repeating the research on a large-scale dataset that contains real-world chart images;
5. Increase the number of supported chart types.

## 6. Conclusions

This paper focuses on chart data extraction using low-level features. To our knowledge, this is the first report of an algorithm whose core process is shared among five chart types: pie charts, donut charts, sunburst diagrams, heatmaps, and waffle charts. In this work, a new, fully automatic low-level algorithm that uses a raster image as input and generates an object-oriented structure of the chart of that image is presented. The main novelty of the proposed approach is in chart processing on binary images instead of commonly used pixel counting techniques. Moreover, a synthetic, large-scale dataset containing 120,000 chart images organized into 20 categories is created and made publicly available to the research community [https://doi.org/10.6084/m9.figshare.19524844.v1 (accessed on 6 April 2022)]. The achieved average data extraction accuracy on the synthetic dataset can be considered state-of-the-art within multiple error rate groups. When discussing the 5% error rate, 99% of tested chart images are within the threshold value. The sunburst diagrams, heatmaps, and waffle charts have never been explored before, and this is the first research that presents comparable numbers. Real-world chart images were also tested, but the decrease in performance is noticeable due to diversity in the design of chart images and the small dataset size. Despite the limitations, these findings are valuable in light of future research on chart data extraction.

An application and experimental analysis of the suitability of the proposed algorithm on real-world chart images is one of the most important subjects for further research. Currently, no real-world chart dataset is publicly available. The research presented in this work is a good starting point for further research, where the focus will be on the creation of real-world datasets and the increase in the number of supported chart types.

In our future work, we will continue studying the use of binary images in other chart types. We believe that the presented algorithm with binary masks can be used with any chart type. With the increased number of supported chart types, we will refine our model so that it can eventually extract data from general chart images and not be limited by design.

## Figures and Tables

**Table 1:** A short summary of presented related work.

Ref.	Year	Bar [%]	Line [%]	Pie [%]	Scatter [%]	Dataset
Yang et al. [19]	2006	-	-	-	-	115
Savva et al. [22]	2011	79	-	62	-	105
Mishchenko and Vassilieva [9]	2011	-	-	67	-	300
Al-Zaidy et al. (2015) [26]	2015	83	-	-	-	18
Al-Zaidy et al. (2016) [27]	2016	86	-	-	-	300
Al-Zaidy et al. (2017) [28]	2017	98	-	-	-	213
Cliche et al. [20]	2017	-	-	-	84	50
Jung et al. [18]	2017	-	-	-	-	35
Dai et al. [29]	2018	83	-	-	-	59
Balaji et al. [23]	2018	79	-	79	-	-
Paramita De [24]	2018	-	-	99	-	200
Choi et al. [14]	2019	99	72	92	-	300
Liu et al. [25]	2019	79	-	88	-	-
Rane et al. [30]	2021	72	-	-	-	516
Zhou et al. [31]	2021	85	-	-	-	330
Chen and Zhao [21]	2021	91	89	-	90	3600

The symbol “-” denotes that authors did not report results of data extraction.

**Table 2:** The comparison of the datasets from related work.

Related Work	Liu et al. [32]	Siegel et al. [33]	Savva et al. [22]	Kafle et al. [34]	Jobin et al. [15]	Davila et al. (2019) [35]	Davila et al. (2020) [36]	Our Proposed
Used for	CC	CC, CTP, CDE	CC, CTP, CDE	CDE	CC	CC, CTP, CDE	CC, CTP, CDE	CDE
Chart type								
Area	-	-	+	-	+	-	+	+
Bar	+	+	+	+	+	+	+	+
Block	-	-	-	-	+	-	-	-
Box	-	-	-	-	+	+	+	+
Bubble	-	-	-	-	+	-	-	+
Confusion	-	-	-	-	+	-	-	-
Contour	-	-	+	-	+	-	-	-
Donut	-	-	-	-	-	+	-	+
Flow	+	+	-	-	+	-	-	-
Heatmap	-	-	-	-	+	-	+	+
Histogram	-	-	-	-	+	-	-	+
Line	+	+	+	-	+	+	+	+
Manhattan	-	-	-	-	-	-	+	-
Map	-	-	-	-	+	-	+	-
Pareto	-	-	+	-	+	-	-	-
Pie	-	-	+	-	+	+	+	+
Polar	-	-	-	-	+	-	-	-
Radar	-	-	+	-	+	-	-	-
Scatter	+	+	+	-	+	+	+	+
Sunburst	-	-	-	-	-	-	-	+
Surface	-	-	-	-	+	-	+	-
Table	+	-	+	-	+	-	-	+
Tree	-	-	-	-	+	-	-	-
Vector	-	-	-	-	+	-	-	-
Venn	-	-	-	-	+	-	+	-
Waffle	-	-	+	-	-	-	-	+
Approximate	5000	30,000	2000	300,000	30,000	200,000	25,000	120,000

Symbol “+” states that the chart type is included in the dataset while symbol “-” denotes that authors did not report using this chart type in their dataset. The abbreviations stand for CC—chart classification, CTP—chart text processing, and CDE—chart data extraction.

**Table 3:** The details of our dataset.

Chart Type	Chart Sub-Type	Number of Images			
		Train		Test	
		Simple	Complex	Simple	Complex
Area	-	2000	2000	1000	1000
Bar	Basic horizontal	2000	2000	1000	1000
	Basic vertical	2000	2000	1000	1000
	Grouped horizontal	2000	2000	1000	1000
	Grouped vertical	2000	2000	1000	1000
	Stacked horizontal	2000	2000	1000	1000
	Stacked vertical	2000	2000	1000	1000
Box	Horizontal	2000	2000	1000	1000
	Vertical	2000	2000	1000	1000
Bubble	-	2000	2000	1000	1000
Donut	-	2000	2000	1000	1000
Heatmap	-	2000	2000	1000	1000
Histogram	Horizontal	2000	2000	1000	1000
	Vertical	2000	2000	1000	1000
Line	-	2000	2000	1000	1000
Pie	-	2000	2000	1000	1000
Scatter	-	2000	2000	1000	1000
Sunburst	-	2000	2000	1000	1000
Table	-	2000	2000	1000	1000
Waffle	-	2000	2000	1000	1000
Total		40,000	40,000	20,000	20,000

Symbol “-” states that the chart type does not include any sub-type.

**Table 4:** Chart data extraction accuracy for our synthetic dataset.

Chart Type	Error Threshold Value			
	0 < err < 1.25	1.25 < err < 2.5	2.5 < err < 5	5 < err < 10
Pie	93 (93.0%)	5 (5.0%)	2 (2.0%)	0 (0.0%)
Donut	94 (94.0%)	5 (5.0%)	1 (1.0%)	0 (0.0%)
Sunburst	92 (92.0%)	7 (7.0%)	1 (1.0%)	0 (0.0%)
Heatmap	97 (97.0%)	1 (1.0%)	2 (2.0%)	0 (0.0%)
Waffle	91 (91.0%)	4 (4.0%)	3 (3.0%)	2 (2.0%)
Sum (Average)	467 (93.4%)	22 (4.4%)	9 (1.8%)	2 (0.4%)

The first number in a table is a number of images, and the second number (shown in parenthesis) is percentage.

**Table 5:** Chart data extraction accuracy for ICDAR synthetic dataset.

Chart Type	Error Threshold Value			
	0 < err < 1.25	1.25 < err < 2.5	2.5 < err < 5	5 < err < 10
Pie	94 (94.0%)	5 (5.0%)	1 (1.0%)	0 (0.0%)
Donut	92 (92.0%)	6 (6.0%)	2 (2.0%)	0 (0.0%)
Sum (Average)	186 (93.0%)	11 (5.5%)	3 (1.5%)	0 (0.0%)

The first number in a table is a number of images, and the second number (shown in parenthesis) is percentage.

**Table 6:** Chart data extraction accuracy for pie chart.

Ref.	Year	Accuracy [%]	Dataset
Savva et al. [22]	2011	62	53
Mishchenko and Vassilieva [9]	2011	67	300
Balaji et al. [23]	2018	79	-
Paramita De [24]	2018	99	100
Choi et al. [14]	2019	92	100
Liu et al. [25]	2019	88	-
Proposed algorithm	2022	98	100

The symbol “-” denotes that authors did not report dataset size.

**Table 7:** Chart data extraction accuracy for real-world chart images. The images are collected using Google search.

Chart Type	Error Threshold Value			
	0 < err < 1.25	1.25 < err < 2.5	2.5 < err < 5	5 < err < 10
Pie	5 (25.0%)	3 (15.0%)	7 (35.0%)	0 (0.0%)
Donut	4 (20.0%)	1 (5.0%)	7 (35.0%)	2 (10.0%)
Sunburst	0 (0.0%)	3 (15.0%)	5 (25.0%)	3 (15.0%)
Heatmap	2 (10.0%)	4 (20.0%)	6 (30.0%)	0 (0.0%)
Waffle	0 (0.0%)	1 (5.0%)	6 (30.0%)	3 (15.0%)
Sum (Average)	11 (11.0%)	12 (12.0%)	31 (31.0%)	8 (8.0%)

The first number in a table is a number of images, and the second number (shown in parenthesis) is percentage.

## Data Availability

Data available in a publicly accessible repository. The data presented in this study is openly available in FigShare at https://doi.org/10.6084/m9.figshare.19524844.v1 (accessed on 13 April 2022).
